# Early Diagnosis of* Helicobacter pylori* Infection in Vietnamese Patients with Acute Peptic Ulcer Bleeding: A Prospective Study

**DOI:** 10.1155/2017/3845067

**Published:** 2017-01-04

**Authors:** Duc Trong Quach, Mai Ngoc Luu, Toru Hiyama, Thuy-HuongThi To, Quy Nhuan Bui, Tuan Anh Tran, Binh Duy Tran, Minh-Cong Hong Vo, Shinji Tanaka, Naomi Uemura

**Affiliations:** ^1^Department of Internal Medicine, University of Medicine and Pharmacy, Ho Chi Minh, Vietnam; ^2^Department of Gastroenterology, Gia Dinh People's Hospital, Ho Chi Minh City, Vietnam; ^3^Health Service Center, Hiroshima University, Higashihiroshima, Japan; ^4^Department of Endoscopy, Gia Dinh People's Hospital, Ho Chi Minh City, Vietnam; ^5^Department of Endoscopy, Hiroshima University Hospital, Hiroshima, Japan; ^6^Department of Gastroenterology and Hepatology, National Center for Global Health and Medicine, Ichikawa, Japan

## Abstract

*Aims*. To investigate* H. pylori* infection rate and evaluate a combined set of tests for* H. pylori *diagnosis in Vietnamese patients with acute peptic ulcer bleeding (PUD).* Methods*. Consecutive patients with acute PUB were enrolled prospectively. Rapid urease test (RUT) with 3 biopsies was carried out randomly. Patients without RUT or with negative RUT received urea breath test (UBT) and serological and urinary* H. pylori* antibody tests.* H. pylori* was considered positive if RUT or any noninvasive test was positive. Patients were divided into group A (RUT plus noninvasive tests) and group B (only noninvasive tests).* Results*. The overall* H. pylori* infection rate was 94.2% (161/171). Groups A and B had no differences in demographic characteristics, bleeding severity, endoscopic findings, and proton pump inhibitor use.* H. pylori-*positive rate in group A was significantly higher than that in group B (98.2% versus 86.7%, *p* = 0.004). The positive rate of RUT was similar at each biopsy site but significantly increased if RUT results from 2 or 3 sites were combined (*p* < 0.05).* Conclusions*.* H. pylori* infection rate in Vietnamese patients with acute PUB is high. RUT is an excellent test if at least 2 biopsies are taken.

## 1. Introduction

*Helicobacter pylori* (*H. pylori) *is one of the leading causes of peptic ulcer disease, which may lead to severe complications such as peptic ulcer bleeding (PUB) or perforation. Diagnosis and successful* H. pylori *eradication have been shown to prevent recurrent PUB [1, 2]. Recently, the importance of early diagnosis and eradication of* H. pylori *during admission period of patients with PUB has been further emphasized because the rate of recurrent PUB has been reduced with such strategy [3]. A recent study reported that up to 40% of PUB patients with* H. pylori* infection did not receive* H. pylori *eradication therapy and were lost to follow-up if diagnostic tests were performed after discharge [4]. Another study showed that less than 50% of patients with PUB received* H. pylori *testing and less than 10% had any* H. pylori *testing after discharge [5].

The fact that many diagnostic tests for* H. pylori *are not so highly sensitive and have high false-negative value during acute bleeding period creates a clinical challenge [6–8]. As a consequence, the diagnosis of* H. pylori *infection during acute bleeding situations has been reported to be lower than the true number [9]. A combination of diagnostic tests may help to document more correctly the prevalence of* H. pylori *infection among patients with PUB. The rates of* H. pylori* infection among Vietnamese patients with upper gastrointestinal symptoms were reported of 55.5%–65.5% in previous studies [10, 11]. But there have been no studies on the prevalence of* H. pylori *in Vietnamese patients with acute PUB. The Maastricht IV consensus recommended that* H. pylori *eradication treatment should be started at reintroduction of oral feeding in cases of bleeding ulcer as delaying treatment after discharge leads to reduced compliance or loss to follow-up without receiving treatment [12]. In addition, validated IgG antibody can be used to diagnose* H. pylori* infection in patients with no prior history of* H. pylori *eradication. In clinical practice, therefore, only one positive test is enough to initiate* H. pylori* eradication therapy in patients with bleeding peptic ulcers. In this study, we imitate the real clinical scenario of peptic ulcer bleeding, when sometimes invasive diagnostic tests for* H. pylori *(rapid urease test, histology, and culture) could not be done. This study aimed to assess the prevalence of* H. pylori *infection and evaluate the role of a combined set of tests for* H. pylori *early diagnosis in Vietnamese patients with acute PUB. We did not assess the sensitivity and specificity of each diagnostic test, which has been very nicely reported in a previous meta-analysis [7], but try to identify the testing strategy which has the high possibility of early detecting* H. pylori *infection in this setting.

## 2. Methods

### 2.1. Patients

From August 2015 to April 2016, consecutive patients aged ≥ 18 years, hospitalized with acute upper gastrointestinal bleeding, and endoscopically diagnosed with gastric and/or duodenal ulcers at Gia Dinh People's Hospital, Ho Chi Minh, Vietnam, were recruited prospectively. Patients with prior history of gastrectomy were excluded. The study protocol was approved by the Ethics Committee of Gia Dinh People's Hospital.

### 2.2. Data Collection

Patients admitted with the presentation of upper gastrointestinal bleeding (hematemesis, melena, or hematochezia) were resuscitated and prepared for upper gastrointestinal endoscopy. During endoscopy, the presence of blood in the upper gastrointestinal tract and the recent stigmata of bleeding ulcers were recorded. Endoscopic intervention was performed if high-risk lesions (*i.e.*, spurting or oozing, visible vessels, or blood clot adhered to the ulcer base) were identified. When patients were stabilized after endoscopy, they were asked to fill out a questionnaire that included questions regarding demographic data, history of administration of nonsteroidal anti-inflammatory drugs (NSAIDs), proton pump inhibitors (PPIs), and antibiotics, peptic ulcer, and* H. pylori* eradication therapy. The hemodynamic instability at admission and preendoscopic PPI use were also recorded.

### 2.3. *Helicobacter pylori* Tests

*H. pylori *tests which were used in this study included rapid urease test (RUT), urea breath test (UBT), and serological and urinary* H. pylori* antibody tests. Patients without RUT or with negative RUT result then received noninvasive tests, which included UBT, and serological and urinary* H. pylori* antibody tests.

#### 2.3.1. Rapid Urease Test

RUT (PyloriTek, Serim Research Corp., Elkhart, Ind., USA) was carried out randomly (2 : 1 basis). Three biopsy specimens were taken and tested separately from each patient: the midantrum in the greater curvature (site 1), the lower corpus (site 2), and the midcorpus (site 3) in the greater curvature. The results were read after 1 hour as recommended by the manufacturer and considered to be positive when blue spots appeared over at least one specimen to the same color level as that of the positive control and negative when color changes were absent.

#### 2.3.2. Urea Breath Test

UBT was performed once patients were allowed to drink and eat again. It was done using a commercially available diagnostic method (Helicobacter Test INFAI, INFAI GmbH, Cologne, Germany), with ^13^C labelled urea to detect urease activity, indicating the presence of* H. pylori* [13, 14].

#### 2.3.3. Serological* Helicobacter pylori* Antibody Test

Qualitative serological detection of specialized human immunoglobulin G (IgG) antibodies to* H. pylori* was done with* Instant-View*®* H. pylori Rapid Test* (Alfa Scientific Designs Inc., Poway, CA, USA).

#### 2.3.4. Urinary* Helicobacter pylori* Antibody Test

Urinary* H. pylori* antibody status was determined with a rapid urinary test (Rapirun® H. Pylori Antibody Stick, Otsuka Pharmaceutical Co., Ltd, Tokyo, Japan). The test measures human IgG antibody against* H. pylori *in urine using the principle of immunochromatography [10].

#### 2.3.5. Definition of* Helicobacter pylori-*Positive

*H. pylori* infection was diagnosed when RUT was positive at any biopsy site or at least one other* H. pylori *test was positive.

### 2.4. Statistical Analysis

Baseline characteristics were presented as mean ± standard deviation (SD) for continuous variables and as a frequency (percentage) for all variables. The recruited patients were divided into 2 groups according to the tests that used for* H. pylori *infection diagnosis: group A (RUT plus noninvasive* H. pylori *tests) and group B (only noninvasive* H. pylori* tests).

The chi-square and two-tailed Fisher's exact test were performed to evaluate whether the demographic, clinical characteristics, endoscopic findings, and* H. pylori *infection rate were different between groups A and B. In addition, McNemar's test was used to assess the differences in* H. pylori *infection rates among different RUT biopsy sites.

## 3. Results

### 3.1. Patient Characteristics

During the study period, there were 177 consecutive patients with acute PUB admitted to Gia Dinh People's Hospital. Six patients with prior history of gastrectomy were excluded. The mean age of 171 patients recruited in the study was 55.4 ± 17.3. Forty-two (24.6%) patients had prior history of gastroduodenal ulcers, including six with perforated peptic ulcer managed by simple suture operation. Twelve (7.0%) patients had been diagnosed with* H. pylori *infection and received eradication therapy. Forty-three (25.1%) patients used NSAIDs before admission. The number of patients using PPIs within 2 weeks and antibiotics within 4 weeks before admission was 28 (16.4%) and 13 (7.6%), respectively. Preendoscopy high-dose proton pump inhibitors were prescribed in 133 (77.8%) patients. The detailed characteristics of patients in this study are presented in Table 1.

### 3.2. Endoscopic Setting and Findings

Upper gastrointestinal endoscopy was performed within 12 hours in 138 (80.7%), within 12–24 hours in 16 (9.4%), and within more than 24 hours after admission in 17 (9.9%) patients. Blood was present in the gastrointestinal tract in 75 (43.9%) patients. The proportions of gastric ulcer, duodenal ulcer, and gastroduodenal ulcer were 42.7%, 49.1%, and 8.2%, respectively. The high-risk stigmata of ulcer in this study included spurting/oozing (11.7%), visible vessel (14.6%), and adherent clot (32.7%) (Table 2).

### 3.3. *Helicobacter pylori* Tests

RUT was performed in 111 patients (group A) and not performed in 60 patients (group B). No patients in group A required intervention for bleeding related to gastric mucosal biopsy for RUT. There were 8 patients in group A and 4 patients in group B who had prior history of* H. pylori *eradication therapy.

**Figure 1:**
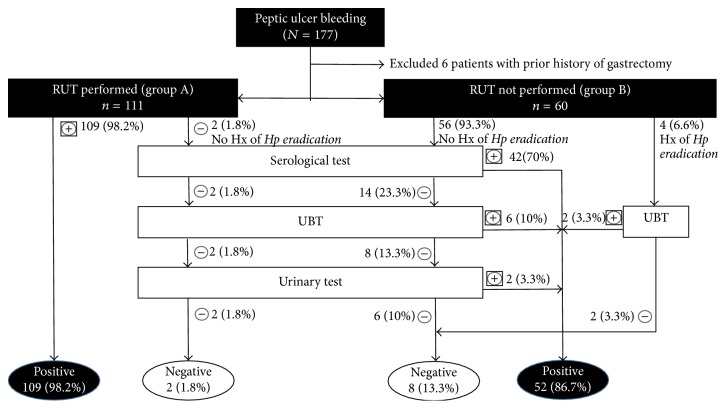
*H. pylori *testing in Vietnamese patients with acute peptic ulcer bleeding.

All patients with* H. pylori *infection in group A were diagnosed by RUT. Additional testing with serological and urinary tests and UBT identified no additional* H. pylori* infected patients (Figure 1). Patients without RUT or with negative RUT result then received noninvasive tests including serological and urinary tests and UBT. In group A, RUT was positive in 98.2% (109/111) patients including 6 of the 8 who had prior history of* H. pylori *eradication therapy. Two patients with negative RUT (both had no prior history of* H. pylori *eradication therapy) were tested with serological and urinary tests and UBT, which were all negative. In group B, 56 (93.3%) patients without prior history of* H. pylori *eradication therapy were tested with serological and urinary tests and UBT, which showed 50 patients with* H. pylori *infection. Four (6.6%) patients had prior history of* H. pylori *eradication therapy, and 2 of them were* H. pylori-*positive.* H. pylori *infection rate in group A was significantly higher than that in group B (98.2% versus 86.7%, *p* = 0.004) while the other characteristics between the 2 groups were not significantly different (Table 3).

**Figure 2:**
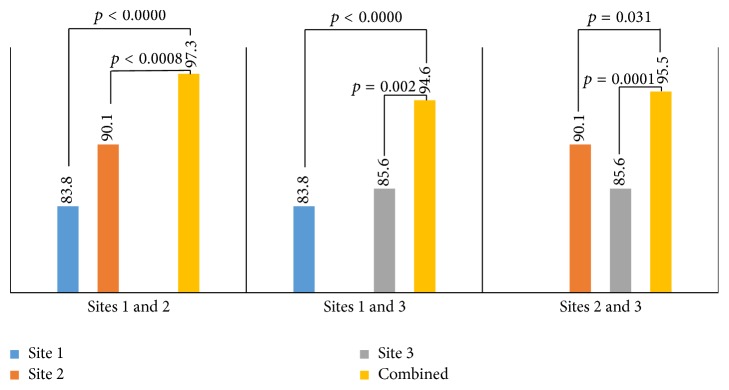
RUT results from single specimen versus combined specimens from two biopsy sites.

The detection rates of* H. pylori *infection by RUT with each single biopsy taken from site 1 (83.8%), site 2 (90.1%), and site 3 (85.6%) were not significantly different (*p* = 0.284) (Table 4). The detection rates by combined results from 2 biopsy sites: site 1 and site 2 (97.3%), site 1 and site 3 (94.6%), and site 2 and site 3 (95.5%) were also not different from each other but significantly increased when compared with each single biopsy site (Figure 2). The detection rate combined result from 3 biopsy sites was the highest, 98.2%.

## 4. Discussion

Accurately detecting* H. pylori* and subsequently eradicating the organism in infected patients with PUB are important to avoid recurrent bleeding. The recurrence rate has been reported in only 3% of the patients who received eradication treatment but 20% in those treated with antisecretory noneradicating therapy (without subsequent long-term maintenance antisecretory therapy) [15, 16]. The challenge is that many diagnostic tests including RUT, histology, and culture have been reported to have low sensitivity in patients with acute PUB [7]. In a metaregression analysis, the infection rate was significantly higher when diagnostic testing was delayed until at least 4 weeks following the bleeding event, suggesting that retesting* H. pylori *at a later time in PUB patients with initially negative test results was necessary [9]. However, late diagnosis of* H. pylori *infection in PUB patients leads to significant number of those with* H. pylori *infection who did not receive eradication therapy [4]. Therefore, increasing the sensitivity of tests for early diagnosis of* H. pylori *infection during admission period of PUB is crucial.

Regarding first-choice diagnostic tests for* H. pylori*, the American College of Gastroenterology recommended biopsy-based tests [17]. Among these, RUT is the most popular test in clinical practice but has high variable number of false-negative results according to a meta-analysis [7]. Sixteen studies included in this study showed a high degree of heterogeneity with sensitivities ranging between 0.41 and 0.94. When subanalysis of the biopsy sites for RUT was performed with only samples obtained from both the antrum and corpus which were considered, heterogeneity among sensitivities substantially decreased and pooled sensitivity increased. The results of our study help clarify this issue. The number and location of biopsy specimens are among key factors to increase RUT sensitivity. The detection rates of* H. pylori* infection by RUT with specimen taken from each biopsy site in our study were not different, but significantly increased when specimens from 2 biopsy sites were combined (Table 4 and Figure 2). The combined result from 3 biopsy sites helps to detect even more patients with* H. pylori* infection, showing that negative RUT result from 2 biopsy sites is still not enough to exclude* H. pylori *infection and additional diagnostic tests or delaying diagnostic tests should be done.

Because some studies have found that all endoscopic-based diagnostic tests have a lower sensitivity in patients with acute PUB [7], it has been recommended that noninvasive methods should be used in patients who have negative result of endoscopic-based diagnostic tests [17]. Two patients in group A who had negative RUT result were performed serological test, UBT, and urinary test and had negative results. Therefore, all patients with* H. pylori* infection in group A were diagnosed by RUT and the total infection rate in this group was 98.2%. As the specificity of RUT during PUB is very high [6, 7], this figure likely represents for the true prevalence of* H. pylori* in Vietnamese patients with PUB. This is truly first data on Vietnamese population so far. Previous studies in other populations showed a significant lower prevalence of* H. pylori *in PUB patients. In European studies, the prevalence of* H. pylori* in PUB patients was lower than that in patients with uncomplicated peptic ulcer disease, varying from 43% to 56%, and was explained by NSAIDs use [18]. In our study, the rate of NSAIDs use was 25.1%. In addition to this, a significant number of patients had prior history of NSAIDs use and were also infected with* H. pylori. *These 2 factors have been confirmed as independent risk factors for the development of peptic ulcer disease, associated bleeding, and the risk was significantly augmented when both factors presented [19]. Therefore, patients with PUB who have a single negative* H. pylori *test and a prior history of NSAIDs use should not be simply referred to as NSAIDs-induced PUB. Further diagnostic tests should be done to detect* H. pylori *infection.

The difference in infection rate of* H. pylori* between our study and other studies may be explained by many reasons, such as the different infection rate of* H. pylori* in each population, the differences in biopsy protocol for RUT, the endoscopy timing, and the types of RUT kit among studies. Many other studies used CLOtest® while we used PyloriTek® [6, 7]. Previous studies showed that results of the PyloriTek at 1 hour and CLOtest at 24 hours are comparable [20, 21], but there have been no direct comparison of the 2 kits in the setting of PUB patients, which is a research question of our future study.

Although biopsy-based tests for* H. pylori *are recommended as preferred diagnostic tests in PUB patients, they could not be performed in some situations because endoscopic treatment may have already made the procedure too long or patients were not hemodynamically stable during the procedure. In some developing countries, upper gastrointestinal endoscopy is performed under local anesthesia and patients' cooperation with prolonged procedure may be difficult. Therefore, noninvasive tests for* H. pylori *diagnosis are still required. According to a meta-analysis,* H. pylori *infection was accurately diagnosed by serological test in patients with PUB [7]. In our study, the test was the first choice among noninvasive diagnostic tests as it is widely available. In addition, UBT was used if serological test was negative. And urinary test was also used as it was locally validated in Vietnam with acceptable accuracy [10]. Although the specificity of this noninvasive approach is not as good as that of RUT in the setting of recent gastrointestinal bleeding [7], we try to combine this set of noninvasive tests in order to early detect all possibly* H. pylori *infection during admission time. But in spite of combining these three noninvasive tests, the total positive rate of* H. pylori *infection in group B was still significantly lower than that in group A. As other characteristics between the 2 groups were not different (Table 3), this result clearly shows that RUT with at least 2 biopsy sites is an important test for* H. pylori *diagnosis in patients with PUD.

Our study has several weak points. First, number of patients included in this study was relatively limited. Studies with much more patients are needed to verify our results. Second, the definition of* H. pylori-*positive was based on only one diagnostic test. One false-positive finding may affect the diagnosis. And third, diagnostic tests for* H. pylori* infection such as histological, cultural, and fecal antigen tests were not included in the design of this study. If these tests were included in this study, the results might differ.

In conclusion, our study shows that the* H. pylori* infection rate in Vietnamese patients with acute PUB is high. RUT is an excellent test for detecting* H. pylori* infection in this setting if at least 2 specimens from different biopsy sites are taken. In case that RUT is not performed, late* H. pylori *retesting may be required even when a combined set of noninvasive tests have shown negative results.

## Figures and Tables

**Table 1:** Demographics and clinical characteristics (*n* = 171).

Demographics and clinical characteristics	*n* (%)
Age (mean ± SD)	55.4 ± 17.3
Male	131 (76.6)
Medical use before admission	
NSAIDs	43 (25.1)
PPIs ≤ 2 weeks	28 (16.4)
Antibiotics ≤ 4 weeks	13 (7.6)
Prior history of peptic ulcers	42 (24.6)
Prior history of *H. pylori *eradication	12 (7.0)
Presenting symptoms	
Hematemesis	88 (51.5)
Melena	77 (45)
Hematochezia	6 (3.5)
Preendoscopic PPIs use	
High-dose, intravenous	133 (77.8)
Low-dose, intravenous	8 (4.6)
Oral	3 (1.8)
None	27 (15.8)
Hemodynamic instability at admission (heart rate > 100 beats per minute and/or systemic blood pressure < 100 mmHg)	47 (27.5)
Transfusion requirement	47 (27.5)

**Table 2:** Endoscopic setting and findings (*n* = 171).

Endoscopic setting and findings	*n* (%)
Timing	
<12 h	138 (80.7)
12–24 h	16 (9.4)
>24 h	17 (9.9)
Presence of blood in gastrointestinal tract	75 (43.9)
Location of ulcer	
Gastric	73 (42.7)
Duodenal	84 (49.1)
Gastric and duodenal	14 (8.2)
Endoscopic stigmata	
Spurting	1 (0.6)
Oozing	19 (11.1)
Visible vessel	25 (14.6)
Adherent clot	56 (32.7)
Red spot	17 (9.9)
Clean-based	53 (31.0)

**Table 3:** The characteristics of patients in groups A and B.

Characteristics	Group A (*n* = 111) *n* (%)	Group B (*n* = 60) *n* (%)	*p*
Age (mean ± SD)	54.7 ± 17.6	56.1 ± 16.8	0.696
Male	84 (75.7)	47 (78.3)	0.695
Prior history of gastroduodenal ulcers	28 (25.2)	14 (23.3)	0.784
Prior history of *H. pylori *eradication	8 (7.2)	4 (6.7)	1.000
Medical use before admission			
NSAIDs	28 (25.2)	15 (25.0)	0.974
PPIs within 2 weeks	21 (18.9)	7 (11.7)	0.221
Antibiotics within 4 weeks	10 (9.0)	3 (5.0)	0.547
Hemodynamic instability at admission (heart rate > 100 beats per minute or systemic blood pressure < 100 mmHg)	29 (26.1)	18 (30.0)	0.219
Preendoscopic PPIs use			
High-dose, intravenous	89 (80.2)	44 (73.3)	0.136
Low-dose, intravenous	6 (5.4)	2 (3.3)	
Oral	3 (2.7)	0 (0)	
None	13 (11.7)	14 (23.3)	
Timing of endoscopy			
<12 h	84 (75.7)	54 (90.0)	
12–24 h	13 (11.7)	3 (5.0)	0.077
>24 h	14 (12.6)	3 (5.0)	
Presence of blood in endoscopy	52 (46.8)	23 (38.3)	0.284
Location of ulcer			
Gastric	49 (44.1)	24 (40.0)	0.763
Duodenal	54 (48.6)	30 (50.0)	
Gastric and duodenal	8 (7.2)	6 (10.0)	
Endoscopic stigmata			
Spurting	1 (0.9)	0 (0)	0.448
Oozing	13 (11.7)	6 (10.0)	
Visible vessel	20 (18.0)	5 (8.3)	
Adherent clot	33 (29.7)	23 (38.3)	
Red spot	12 (10.8)	5 (8.3)	
Clean-based	32 (28.8)	21 (35.0)	
*H. pylori-*positive rates	109 (98.2)	52 (86.7)	0.004

**Table 4:** RUT results with specimens taken from different biopsy sites.

Biopsy sites	*H. pylori-*positive cases *n* (%)
Single biopsy site	
1 (midantrum, greater curvature)	93 (83.8)
2 (low-corpus, greater curvature)	100 (90.1)
3 (midcorpus, greater curvature)	95 (85.6)
Two biopsy sites (combined results)	
1 & 2	108 (97.3)
1 & 3	105 (94.6)
2 & 3	106 (95.5)
Three biopsy sites (combined result)	
1, 2, and 3	109 (98.2)
